# ‘This is the last episode’: the association between problematic binge‐watching and loneliness, emotion regulation, and sleep‐related factors in poor sleepers

**DOI:** 10.1111/jsr.13747

**Published:** 2022-10-17

**Authors:** Valentina Alfonsi, Giorgia Varallo, Serena Scarpelli, Maurizio Gorgoni, Maria Filosa, Luigi De Gennaro, Alessandro Musetti, Christian Franceschini

**Affiliations:** ^1^ Department of Psychology Sapienza University of Rome Rome Italy; ^2^ Department of Medicine and Surgery University of Parma Parma Italy; ^3^ IRCCS Fondazione Santa Lucia Rome Italy; ^4^ Department of Humanities, Social Sciences and Cultural Industries University of Parma Parma Italy

**Keywords:** binge‐viewing, clinical psychology, daytime sleepiness, emotional dysregulation, isolation, sleep quality

## Abstract

Evidence on the relation between binge‐watching and sleep quality is still scarce and inconsistent and none has taken into account both the healthy and pathological dimensions of the phenomenon. This study aimed at filling this gap by investigating both aspects in healthy participants with high and low sleep quality. Further, we aimed at identifying sociodemographic, psychological and sleep‐related determinants of problematic binge‐watching in poor sleepers. We first conducted independent comparisons between good (*n* = 253) and poor sleepers (*n* = 209) on different binge‐watching symptoms and motives, assessed through ‘Binge‐watching Engagement and Symptoms’ and ‘Watching TV Series Motives’ questionnaires, respectively. Then, we focused on the problematic aspects of binge‐watching in poor sleepers, investigating the role of emotion regulation, loneliness, and sleep‐related factors using hierarchical multiple regressions. Comparisons between the two groups revealed a greater extent of binge‐watching behaviour (*t* = −2.80, *p* = 0.005) and greater use of this practise to cope with negative emotions (*t* = −4.17, *p* < 0.001) in poor sleepers. In addition, hierarchical multiple regressions showed that gender (β = −0.166, *p* = 0.008), alcohol consumption (β = −0.135, *p* = 0.035), emotional dysregulation (β = 0.260, *p* = 0.001; β = 0.298, p < 0.001), feelings of loneliness (β = 0.159, *p* = 0.029; β = 0.199, *p* = 0.003), and daytime sleepiness (β = 0.149, *p* = 0.016) are significant determinants of problematic binge‐watching in this population. In addition to showing for the first time the relationship between sleep quality and different aspects of binge‐watching, our findings indicate that emotional dysregulation, feelings of loneliness, and daytime sleepiness play a key role in determining problematic binge‐watching in poor sleepers, possibly due to the existence of a pathological vicious circle between these factors in poor sleepers.

## INTRODUCTION

Over the last decade, the expansion of video‐on‐demand and the unlimited access to online streaming platforms have significantly contributed to the rapid spread of ‘binge‐watching’, a phenomenon defined as watching multiple episodes of television (TV) series in a single session over an extended period (Walton‐Pattison et al., 2018). Evidence suggests that young adults are more likely to binge‐watch than older people (Jay, 2022) and that women are more prone to binge‐watching (Pittman & Sheehan, 2015; Spruance et al., 2017).

Many studies assessing individual engagement in binge‐watching found a wide range of motives behind this habit. On the one hand, individuals may be driven by entertainment, gratification, social connection, or relaxation (Castro et al., 2021; Panda & Pandey, 2017). On the other hand, people may undertake compulsive viewing of TV series as a coping strategy to deal with their negative emotions and escape from reality (Rubenking & Bracken, 2018; Starosta et al., 2019).

Several studies showed that problematic binge‐watching might have negative psychosocial and health consequences (Chambliss et al., 2017; Riddle et al., 2018). Also, it is a highly immersive experience, which could lead to loss of control (De Feijter et al., 2016; Devasagayam, 2014). These characteristics have led some researchers to emphasise the similarities between binge‐watching and behavioural addictions (Flayelle et al., 2019a). However, this approach may result in an over‐simplification, as well as an over‐pathologising of this heterogeneous and entrenched phenomenon (Billieux et al., 2015).

An alternative approach aimed at disentangling typical and problematic aspects of binge‐watching focused on its underlying processes (Flayelle et al., 2019b; Ort et al., 2021). Accordingly, Flayelle et al. (2019c) developed and validated two innovative questionnaires, assessing different symptoms and motivations behind binge‐watching behaviour. Unsurprisingly, some motivations were associated with problematic binge‐watching (e.g., coping/escapism), whereas others defined high but healthy involvement in TV series (e.g., enrichment).

Coping with loneliness is a common feature of heavy binge‐watchers (Gao et al., 2018; Masur et al., 2014; Starosta & Izydorczyk, 2020), suggesting that it could be used as a coping strategy to deal with dysfunctional moods. Thus, problematic binge‐watching, like other addictive behaviours, can be a strategy to regulate aversive emotions such as loneliness (Flayelle et al., 2019a; Rubenking & Bracken, 2018). A recent study by Starosta et al. (2021) found that difficulties in emotion regulation were significant predictors of problematic binge‐watching.

To date, the only study that explored the association between binge‐watching and sleep outcomes found that higher levels of binge‐watching were related to poorer sleep quality, fatigue, and insomnia symptoms (Exelmans & Van den Bulck, 2017). In turn, poor sleep quality is associated with less ability in emotion regulation and feelings of loneliness. Sleep disruption has a negative effect on cognitive functions, such as cognitive control (Goel et al., 2009; Tucker et al., 2010), which plays a fundamental role in effective emotion regulation (Ochsner & Gross, 2005). Furthermore, self‐reported loneliness is linked to worse sleep quality (Griffin et al., 2020). Besides sleep quality, other sleep‐related factors are associated with both these aspects. The evening chronotype is known to be associated with maladaptive coping strategies (Adan et al., 2017), emotion dysregulation (Antúnez, 2020), and loneliness (Norbury, 2021). Also, preliminary evidence showed that daytime sleepiness is linked to difficulties with emotion dysregulation (Toney, 2019) and feelings of loneliness (Holding et al., 2020). These characteristics, taken together, suggest that poor sleepers might be more prone to problematic binge‐watching through a vicious circle with detrimental effects on both aspects.

Evidence on the relationship between sleep and binge‐watching is still scarce and inconsistent (Exelmans & Van den Bulck, 2017; Oberschmidt, 2017). This study aimed at filling this gap by exploring the following research questions: (a) Do poor and good sleepers amongst the general population exhibit different levels of binge‐watching as a function of the specific symptoms and motives behind this practise? (b) What is the specific explanatory role of sociodemographic, psychological, and sleep‐related factors in explaining problematic binge‐watching in poor sleepers?

According to the multidimensional concept of the binge‐watching phenomenon, we hypothesise a different relationship between sleep quality and binge‐watching as a function of the specific aspects considered. In particular, we expect poor sleepers to exhibit a greater extent of binge‐watching behaviour and to report greater use of binge‐watching as a strategy for coping with negative emotions. Otherwise, no differences between good and poor sleepers are expected concerning healthy and functional aspects of this practise.

Further, we expect to confirm the well‐established determinants of problematic binge‐watching and find loneliness, emotional dysregulation, and diurnal sleepiness as significant contributions to explain the levels of symptomatology related to binge‐watching and its problematic motivations in this population.

## METHODS

### Participants and protocol

This study was conducted through an online survey implemented using the Microsoft Azure platform and shared across various social media and university platforms. The survey took ~20 min to complete and was enabled from December 2020 to January 2021.

After a short section on sociodemographic, clinical and lifestyle information, participants were requested to complete some questionnaires to assess sleep variables, psychological aspects, and binge‐watching behaviours in the web‐based survey.

In accordance with the aim of observing the relationship between sleep quality and binge‐watching within the general Italian population, we excluded participants from other countries and with self‐declared clinical conditions.

All individuals completed the survey after reading and signing an electronic informed consent and declaring to be aged ≥18 years. Participants could withdraw from the study at any time without any justification and no data were saved. No personally identifiable information was collected to guarantee anonymity. Participation was voluntary and without compensation.

The study was conducted following the Declaration of Helsinki and was approved by the local Ethics Committee (CERIP‐ Comitato Etico del Centro di Ricerca e di Intervento Psicologico University of Messina, March 4, 2021, prot. n. 12,106).

### Measures

#### Sociodemographic, clinical and lifestyle information

The short section first collected sociodemographic information (age, gender, nationality, marital status, education level, occupation). Then, some questions investigated the presence of any pre‐existing diagnostic condition (chronic diseases, sleep, or mental disorders) or any current pharmacological treatment that may cause sleep problems (e.g., antihistamine, steroids, hormones, etc.). Finally, we collected information on sleep habits (i.e., naps, bedtime and wake‐up time at the weekend, evening electronic device use, dark mode organic light‐emitting diode [OLED] screen use), drinking, and smoking habits.

#### Sleep measures

1. Pittsburgh Sleep Quality Index (PSQI) (Curcio et al., 2013): a self‐reported questionnaire to assess subjective sleep quality. The measure consists of 19 items, resulting in seven subscales (subjective sleep quality, sleep latency, sleep duration, habitual sleep efficiency, sleep disturbances, use of sleep medications, daytime dysfunction) and a global sleep quality score. A PSQI global score of >5 indicates a subjectively perceived poor sleep quality.
2. Insomnia Severity Index (ISI) (Castronovo et al., 2016): a self‐reported questionnaire to assess the perceived severity of insomnia (i.e., difficulties falling asleep, staying asleep, waking up too early, and daytime distress related to sleep). The measure consists of seven items. Scores range from 0 to 28 and higher total scores indicate greater insomnia severity. Scores identify ‘no clinically significant insomnia’ (0–7 points), ‘subthreshold insomnia’ (8–14 points), ‘clinical insomnia‐moderate’ (15–21 points), and ‘clinical insomnia‐severe’ (22–28 points).
3. Epworth Sleepiness Scale (ESS) (Vignatelli et al., 2003): a self‐reported questionnaire to assess daytime sleepiness. The measure consists of a list of eight situations in which participants rate their tendency to become sleepy on a scale ranging from 0 (‘no chance to fall asleep’) to 3 (‘high possibility of falling asleep’). Higher total scores indicate greater daytime sleepiness and chance to doze off during daytime activities. Scores identify ‘normal range of sleepiness’ (0–10 points), ‘mild sleepiness’ (11–14 points), ‘moderate sleepiness’ (15–17 points) and ‘severe sleepiness’ (18–24 points).
4. Morningness–Eveningness Questionnaire‐reduced version (MEQr) (Natale et al., 2006): a self‐reported questionnaire to assess the circadian typology, consisting of five items extracted from the MEQ (Mecacci & Zani, 1983). The total score ranges from 4 (‘extreme eveningness’) to 25 (‘extreme morningness’) and total scores >18 define the ‘morning‐type’ and <11 the ‘evening‐type’.

#### Psychological measures

1. Difficulties in Emotion Regulation Scale (DERS) (Sighinolfi et al., 2010): a self‐reported questionnaire to assess the difficulties in emotion regulation in the adult population. The questionnaire consists of 36 items and provides six subscales on different dimensions of emotion regulation: (i) non‐acceptance of emotional responses; (ii) difficulties engaging in goal‐directed behaviour; (iii) impulse control difficulties; (iv) lack of emotional awareness; (v) limited access to emotion regulation strategies; (vi) lack of emotional clarity. Higher total scores indicate greater difficulties in emotion regulation.
2. University of California, Los Angeles (UCLA) Loneliness Scale, version 3 (UCLA LS3) (Boffo et al., 2012): a self‐reported questionnaire to assess feelings of loneliness. The questionnaire consists of 20 items and each item is evaluated on a scale ranging from 1 (‘never’) to 4 (‘always’). Higher total scores indicate greater feelings of loneliness.

#### Binge‐watching measures

1. Binge‐Watching Engagement and Symptoms Questionnaire (BWESQ) (Flayelle et al., 2019c): a self‐reported questionnaire to assess the engagement in watching TV series and the related symptoms. The measure consists of 40 items and each item is evaluated on a scale ranging from 1 (‘strongly disagree’) to 4 (‘strongly agree’). The questionnaire provides seven subscales: engagement (e.g., ‘My family and friends consider me a gold mine of information on TV series’), positive emotions (e.g., ‘Watching TV series is a cause for joy and enthusiasm in my life’), pleasure preservations (e.g., ‘I get really irritated if I get the next few episodes spoiled by anyone’), desire/savouring (e.g., ‘I get really excited when a new episode is released’), binge‐watching (e.g., ‘I usually spend more time watching TV series than planned’), dependency (e.g., ‘I get tense, irritated or agitated when I cannot watch my favourite TV series’) and loss of control (e.g., ‘I watch more TV series than I should’). Higher scores on each subscale indicate higher engagement and symptoms.
2. Watching TV Series Motives Questionnaire (WTSMQ) (Flayelle et al., 2019c): a self‐reported questionnaire to assess the motivations for watching TV series. The measure consists of 22 items and each item is evaluated on a scale ranging from 1 (‘not at all’) to 4 (‘to a great extent’). The questionnaire provides four subscales on different types of motives: coping/escapism (e.g., ‘I watch TV series to pass the time and escape from boredom’), emotional enhancement (e.g., ‘I watch TV series to feel strong emotions like the excitement or the thrill they give me’), enrichment (e.g., ‘I watch TV series to learn or familiarise myself with a new language’), and social motives (e.g., ‘I watch TV series not to be out of touch, because most of my friends do it’). Higher scores on each subscale indicate higher motivation for watching TV series.

### Statistical analyses

Descriptive statistics were used to outline the prevalence of sociodemographic, clinical and lifestyle factors in the whole sample.

To assess the differences in the binge‐watching phenomenon as a function of sleep quality, unpaired Student's *t* tests were performed on each BWESQ scale between good and poor sleepers, categorised according to the PSQI global score cut‐off (PSQI ≤5: ‘good sleepers’ versus PSQI >5: ‘poor sleepers’). The same analysis was carried out on the WTSMQ scales. The chi‐squared test and independent samples Student's *t* tests were used to compare the gender distribution and the age mean of the two groups, respectively. The Bonferroni correction was applied to adjust the *α*‐value for multiple comparisons.

Then, we focused on the BWESQ and WTSMQ scales that had shown higher scores in poor sleepers in the previous analyses, and we conducted hierarchical multiple regressions to observe the best explanatory factors for these pathological scales in that population. For each regression on the BWESQ and WTSMQ scales, the following independent variables were tested: age; gender (male, female); marital status (single, in a relationship); frequency of alcohol consumption; frequency of nicotine consumption; emotional dysregulation (DERS total score); loneliness (UCLA LS3 total score); insomnia symptoms (ISI total score); circadian preference (MEQr total score); daytime sleepiness (ESS total score). The order of entries in hierarchical multiple regression was based on the literature and our specific aims and hypotheses. In particular, we included the sociodemographic variables in the first step, as they were suspected to be strongly related to the dependent variables (Flayelle et al., 2019a). Then, we included the psychological variables in the second step to estimate their predictive value after accounting for sociodemographic variables, given their correlation with binge‐watching behaviour (Starosta et al., 2021). Finally, we included the sleep‐related variables in the third and last step to explore their potential contribution to explaining the problematic aspects of binge‐watching in addition to the sociodemographics and psychological variables.

Collinearity was checked using normal linear regression collinearity diagnostic test. No variance inflation factor ≥5 was observed.

Before applying the above tests, the assumption of normality was checked. For each analysis, a *p* ≤ 0.05 was considered statistically significant.

The statistical analyses were performed using the Statistical Package for Social Sciences (SPSS) version 25.0 and Matlab R2016.

## RESULTS

A total of 528 participants completed the survey and 66 were excluded for several reasons (18 non‐Italian people; 48 people with chronic diseases or drug treatment interfering with sleep). The final sample comprised 462 participants (87.50% of respondents).

### Sociodemographic, clinical and lifestyle characteristics

The characteristics of the participants (total sample, good sleepers, poor sleepers) are shown in Table 1. In short, data from 462 participants revealed that the most represented age range was 18–40 years and most of the participants were females (56.5%). Amongst all respondents, 49.3% were single, 48.9% were married or cohabiting, and a small percentage were divorced/separated/widower (1.8%). Most of the individuals received higher education (61.3%) and were employed (59.3%). Concerning sleep habits, 34% reported daytime napping and bedtime and wake‐up time at the weekend were 12:24 a.m. and 9:11 a.m., respectively. Electronic devices were used in the evening (after 8:30 p.m.) by 94.4% of the participants, of which 47.5% enabled the dark screen mode. Most of the participants were non‐smokers (65.6%) and occasional drinkers (35.7%). Finally, the mean (SE) ISI, ESS and MEQr scores in the overall sample were 5.47 (0.207), 5.53 (0.148) and 15.51 (0.163), respectively.

**TABLE 1:** Sociodemographic, sleep and lifestyle characteristics in the total sample, good sleepers, and poor sleepers

	Total sample (*N* = 462)	Good sleepers (*n* = 253)	Poor sleepers (*n* = 209)	Good sleepers versus Poor sleepers
	*N*°(%)			χ^2^ (*p*)
Age, years				
18–30	212 (45.9)	116 (45.8)	96 (45.9)	0.005 (0.998)
31–40	204 (44.1)	112 (44.3)	92 (44.0)	
≥40	46 (10.0)	25 (9.9)	21 (10.0)	
Gender				
Male	201 (43.5)	114 (45.1)	87 (41.6)	0.549 (0.459)
Female	261 (56.5)	139 (54.9)	122 (58.4)	
Marital status				
Single	228 (49.3)	127 (50.2)	101 (48.3)	3.675 (0.299)
Married/co‐habitant	226 (48.9)	120 (47.4)	106 (50.7)	
Divorced/separated	4 (0.9)	4 (1.6)	0 (0)	
Widower	4 (0.9)	2 (0.8)	2 (1.0)	
Education level				
Until middle School	35 (7.6)	18 (7.1)	17 (8.1)	0.679 (0.954)
High School	283 (61.3)	156 (61.7)	127 (60.8)	
Bachelor's degree	64 (13.8)	33 (13.0)	31 (14.8)	
Master's degree	73 (15.8)	42 (16.6)	31 (14.8)	
PhD/postgraduate school	7 (1.5)	4 (1.6)	3 (1.4)	
Occupation				
Student	160 (34.6)	85 (33.6)	75 (35.9)	1.706 (0.636)
Employed	274 (59.3)	154 (60.9)	120 (57.4)	
Retired	1 (0.2)	1 (0.4)	0 (0)	
Unemployed	27 (5.9)	13 (5.1)	14 (6.7)	
Daytime nap				
Yes	157 (34.0)	76 (30.0)	81 (38.8)	3.876 (0.049)*
No	305 (66.0)	177 (70.0)	128 (61.2)	
Evening electronic device use (after 8:30 p.m.)				
Yes	436 (94.4)	236 (93.3)	200 (95.7)	1.255 (0.263)
No	26 (5.6)	17 (6.7)	9 (4.3)	
Dark mode screen (during evening device use)				
Yes	207 (47.5)	109 (43.1)	98 (49.0)	0.344 (0.558)
No	229 (52.5)	127 (50.2)	102 (51.0)	
Frequency of smoking habits (in the last 2 week)				
Never	303 (65.6)	179 (70.8)	124 (59.3)	10.138 (0.038)*
Rarely or <1 or 2 days	25 (5.4)	13 (5.1)	12 (5.7)	
Some days	28 (6.0)	12 (4.7)	16 (7.7)	
More than half of the days	10 (2.2)	2 (0.8)	8 (3.8)	
Nearly every day	96 (20.8)	47 (18.6)	49 (23.4)	
Frequency of drinking habits (in the last year)				
Never	54 (11.7)	36 (14.2)	18 (8.6)	5.086 (0.279)
Once a month or less	97 (21)	50 (19.8)	47 (22.5)	
2/4 times a month	165 (35.7)	93 (36.8)	72 (34.4)	
2/3 times a week	107 (23.2)	56 (22.1)	51 (24.4)	
At least 4 times a week	39 (8.4)	18 (7.1)	21 (10.0)	
	**Hours**			**t (*p*)**
Bedtime at the weekend:	12:24 a.m.	12:16 a.m.	12:34 a.m.	−2.397 (0.017)*
Wake‐up time at the weekend:	9:11 a.m.	09:13 a.m.	09:08 a.m.	0.456 (0.649)
	**Mean (SE)**			**t (*p*)**
ISI	5.47 (0.207)	3.04 (0.176)	8.41 (0.298)	−16.132 (<0.001)**
ESS	5.53 (0.148)	5.19 (0.196)	5.93 (0.222)	−2.517 (0.012)*
MEQr	15.51 (0.163)	15.78 (0.215)	15.19 (0.248)	1.824 (0.069)

Abbreviations: ESS, Epworth Sleepiness Scale; ISI, Insomnia Severity Index; MEQr, Morningness–Eveningness Questionnaire‐reduced version; SE, standard error.

*Note*: Asterisks indicate significant differences.

### Binge‐watching symptoms and motives in good and poor sleepers

Participants were assigned to the good sleepers (*n* = 253) or poor sleepers (*n* = 209) group according to the global PSQI cut‐off. The gender distribution (χ^2^_1_ = 0.549; *p* = 0.459) and age (t_460_ = −0.130; *p* = 0.897) did not differ significantly between the two categories (good sleepers: 114 males, mean [SD] age 30.03 [8.48] years; poor sleepers: 87 males, mean [SD] age 30.13 [8.36] years).

Table 2 reports the results of the comparisons (unpaired *t* tests) between good and poor sleepers performed on the BWESQ and WTSMQ scales. The two groups exhibited significant difference on the BWESQ‐binge‐watching (t_460_ = −2.80; *p* = 0.005) and WTSMQ‐coping/escapism (t_460_ = −4.17; *p* < 0.001) scales after the Bonferroni correction for multiple comparisons. In both cases, poor sleepers had higher scores than good sleepers.

**TABLE 2:** Results of the comparisons (unpaired *t* tests) between good sleepers (*n* = 253) and poor sleepers (*n* = 209) on the (a) Binge‐Watching Engagement and Symptoms Questionnaire and (b) Watching TV Series Motives Questionnaire scales. Mean and standard errors are reported

	Good sleepers (PSQI ≤5), mean (SE)	Poor sleepers (PSQI >5), mean (SE)	t_460_	*p*
(a) BWESQ scales				
Engagement	14.36 (0.28)	14.87 (0.33)	−1.18	0.24
Positive emotions	10.92 (0.21)	11.29 (0.22)	−1.21	0.22
Pleasure preservation	5.92 (0.14)	6.05 (0.15)	−0.62	0.54
Desire/savouring	13.83 (0.27)	14.65 (0.28)	−2.09	0.04
Binge‐watching	10.64 (0.21)	11.56 (0.26)	−2.80	0.005*
Dependency	7.74 (0.17)	8.09 (0.20)	−1.38	0.17
Loss of control	10.65 (0.23)	11.59 (0.30)	−2.49	0.13
(b) WTSMQ scales				
Social	5.45 (0.12)	5.70 (0.14)	−1.36	0.17
Emotional enhancement	11.06 (0.25)	11.77 (0.27)	−1.93	0.54
Enrichment	10.15 (0.23)	11.00 (0.27)	−2.39	0.02
Coping/escapism	14.65 (0.31)	16.76 (0.40)	−4.17	<0.001*

Abbreviations: BWESQ, Binge‐Watching Engagement and Symptoms Questionnaire; PSQI, Pittsburgh Sleep Quality Index; SE, standard error; WTSMQ, Watching TV Series Motives Questionnaire.

*Note*: Asterisks indicate significant differences.

### Determinants of problematic binge‐watching symptoms and motives in poor sleepers

Results of the hierarchical multiple regressions on the BWESQ‐binge‐watching and WTSMQ‐coping/escapism scales are depicted in Table 3 and Table 4, respectively.

**TABLE 3:** Results of the hierarchical multiple regression for variables predicting Binge‐Watching Engagement and Symptoms Questionnaire‐binge‐watching in poor sleepers (*n* = 200).

BWESQ‐binge‐watching scale	β	t (*p*)	*R* ^2^	Δ*R* ^2^	F change (*p*)	F (*p*)
Step 1			0.031	0.031	1.253 (0.286)	1.253 (0.286)
Gender	−0.021	−0.293 (0.770)				
Age	−0.182	−2.065 (0.040)*				
Smoking frequency	0.020	0.274 (0.784)				
Alcohol frequency	−0.007	−0.099 (0.922)				
Marital status	0.020	0.227 (0.821)				
Step 2			0.164	0.132	15.177 (<0.001)**	5.362 (<0.001)**
Gender	−0.012	−0.176 (0.860)				
Age	−0.116	−1.361 (0.175)				
Smoking frequency	0.009	0.133 (0.894)				
Alcohol frequency	−0.010	−0.140 (0.889)				
Marital status	0.022	0.269 (0.788)				
DERS	0.266	3.619 (<0.001)**				
UCLA LS3	0.180	2.508 (0.013)*				
Step 3			0.179	0.016	1.221 (0.303)	4.133 (<0.001)**
Gender	−0.013	−0.191 (0.849)				
Age	−0.091	−1.026 (0.306)				
Smoking frequency	0.027	0.381 (0.703)				
Alcohol frequency	−0.032	−0.460 (0.646)				
Marital status	0.038	0.453 (0.651)				
DERS	0.260	3.406 (0.001)**				
UCLA LS3	0.159	2.195 (0.029)*				
ISI	0.007	0.105 (0.917)				
MEQr	−0.094	−1.253 (0.212)				
ESS	0.092	1.372 (0.172)				

Abbreviations: BWESQ, Binge‐Watching Engagement and Symptoms Questionnaire; DERS, Difficulties in Emotion Regulation Scale; ESS, Epworth Sleepiness Scale; ISI, Insomnia Severity Index; MEQr, Morningness‐Eveningness Questionnaire‐reduced version; UCLA LS3, University of California, Los Angeles Loneliness Scale version 3.

*Note*: Asterisks indicate significant predictors.

**TABLE 4:** Results of the hierarchical multiple regression for variables predicting Watching TV Series Motives Questionnaire‐coping/escapism in poor sleepers (*n* = 200)

WTSMQ‐coping/escapism scale	β	t (*p*)	*R* ^2^	Δ*R* ^2^	F change (*p*)	F (*p*)
Step 1			0.101	0.101	4.363 (0.001)**	4.363 (0.001)**
Gender	−0.178	−2.573 (0.011)*				
Age	−0.169	−1.984 (0.049)*				
Smoking frequency	0.054	0.761 (0.448)				
Alcohol frequency	−0.104	−1.474 (0.142)				
Marital status	−0.088	−1.041 (0.299)				
Step 2			0.287	0.186	24.967 (<0.001)**	11.020 (<0.001)**
Gender	−0.167	−2.705 (0.007)**				
Age	−0.095	−1.204 (0.230)				
Smoking frequency	0.040	0.622 (0.535)				
Alcohol frequency	−0.106	−1.675 (0.096)				
Marital status	−0.083	−1.081 (0.281)				
DERS	0.303	4.458 (<0.001)**				
UCLA LS3	0.227	3.428 (0.001)**				
Step 3			0.317	0.031	2.819 (0.040)*	8.779 (<0.001)**
Gender	−0.166	−2.700 (0.008)**				
Age	−0.068	−0.839 (0.403)				
Smoking frequency	0.067	1.047 (0.297)				
Alcohol frequency	−0.135	−2.124 (0.035)*				
Marital status	−0.066	−0.858 (0.392)				
DERS	0.298	4.277 (<0.001)**				
UCLA LS3	0.199	3.012 (0.003)**				
ISI	0.007	0.114 (0.910)				
MEQr	−0.102	−1.487 (0.139)				
ESS	0.149	2.421 (0.016)*				

Abbreviation: DERS, Difficulties in Emotion Regulation Scale; ESS, Epworth Sleepiness Scale; ISI, Insomnia Severity Index; MEQr, Morningness‐Eveningness Questionnaire—reduced version; UCLA LS3, University of California, Los Angeles Loneliness Scale version 3; WTSMQ, Watching TV Series Motives Questionnaire.

*Note*: Asterisks indicate significant predictors.

The three‐step hierarchical multiple regression on the BWESQ‐binge‐watching scale (Durbin‐Watson test = 1.986) revealed that at step one, sociodemographic and lifestyle variables (gender, age, smoking frequency, alcohol frequency, and marital status) did not contribute significantly to the regression model (F_5,194_ = 1.253; *p* = 0.286) and accounted for 3.1% of the variation in BWESQ‐binge‐watching. Adding psychological variables (DERS, UCLA LS3) explained an additional 13.2% of the variation and this change in *R* ^2^ was significant (F_2,192_ = 15.177; *p* < 0.001). Lastly, the addition of sleep‐related variables to the regression model explained an additional 1.6% of the variation but this change in *R* ^2^ was not significant (F_3,189_ = 1.221; *p* = 0.303). When all independent variables were included in step three of the regression model, the significant explanatory factors were DERS (β = 0.260; *p* = 0.001) and UCLA LS3 (β = 0.159; *p* = 0.029). Together, the independent variables accounted for 17.9% of the variance in BWESQ‐binge‐watching.

The three‐step hierarchical multiple regression on the WTSMQ‐coping/escapism scale (Durbin‐Watson test = 1.722) revealed that at step one, sociodemographic and lifestyle factors contributed significantly to the regression model (F_5,194_ = 4.363; *p* = 0.001) and accounted for 10.1% of the variation in WTSMQ‐coping/escapism. Introducing psychological factors explained an additional 18.6% of the variation and this change in *R* ^2^ was significant (F_2,192_ = 24.967; *p* < 0.001). Finally, the addition of sleep‐related factors to the regression model explained an additional 3.1% of the variation and this change in *R* ^2^ was also significant (F_3,189_ = 2.819; *p* = 0.040). When all independent variables were included in step three of the regression model, the significant factors were gender (β = −0.166; *p* = 0.008), alcohol frequency (β = −0.135; *p* = 0.035), DERS (β = 0.298; *p* < 0.001), UCLA LS3 (β = 0.199; *p* = 0.003) and ESS (β = 0.149; *p* = 0.016). Together, the independent variables accounted for 31.7% of the variance in WTSMQ‐coping/escapism. The unadjusted regressions of the main independent variables of interests (psychological and sleep) with the primary outcomes of binge watching (i.e., BWESQ‐binge‐watching scale and WTSMQ‐coping/escapism scale) confirmed the explanatory role of loneliness and emotional regulation for both scales and a significant contribution of daytime sleepiness for the WTSMQ‐coping/escapism scale (all statistical results are shown in Table S1 and Table S2).

## DISCUSSION

The present study aimed at evaluating the characteristics of binge‐watching behaviour in participants with good and poor sleep quality taking into account both functional and dysfunctional dimensions of the phenomenon. Specifically, we found excessive binge‐watching and more dysfunctional motives in poor sleepers compared to good sleepers.

Further, we explored the role of sociodemographic, psychological and sleep‐related factors in explaining problematic binge‐watching in poor sleepers. We found that gender, alcohol frequency, emotional dysregulation, feelings of loneliness, and daytime sleepiness were significant determinants.

In agreement with our first hypothesis, good and poor sleepers never exhibited significant differences in binge‐related aspects except for the BWESQ‐binge‐watching and the WTSMQ‐coping/escapism dimensions, which were identified in previous studies as problematic behaviour and motivation, respectively (Flayelle et al., 2019c).

The BWESQ‐binge‐watching scale represents a quantitative measure that reflects the extent of binge‐watching behaviour. Binge‐watching, dependency, and loss of control scales constitute the BWESQ dimensions that correlate with adverse outcomes, such as negative affect and problematic internet use (Flayelle et al., 2019c). On the contrary, other BWESQ dimensions (engagement, positive emotion, desire/savouring, pleasure preservation) show an opposite pattern and reflect genuine interest and involvement in this practise.

Likewise, participants with poor‐sleep quality scored significantly higher than good sleepers only on the pathological scale of the WTSMQ (i.e., WTSMQ‐coping/escapism). This scale reflects the desire to watch TV series to avoid thinking about real‐life problems or to cope with aversive emotional states. Predictably, the coping/escapism dimension is positively associated with problematic binge‐watching factors (i.e., extent of binge‐watching, dependency, loss of control) and other negative outcomes (i.e., problematic Internet use) (Flayelle et al., 2019c).

Overall, our results on sleep quality confirmed and expanded the Flayelle et al. approach, which defines binge‐watching as a complex and multidetermined phenomenon that encompasses both adaptive and maladaptive components.

Concerning our second research question, we confirmed that feelings of loneliness and emotional dysregulation contribute significantly to explaining the extent of binge‐watching behaviour (i.e., BWESQ‐binge‐watching) and the escapism/coping motives (i.e., WTSMQ‐coping/escapism).

Furthermore, we also found that higher levels of daytime sleepiness were related to a greater tendency to watch TV series to escape from reality or cope with stress and negative emotions.

As stated above, these psychological and sleep‐related aspects (i.e., loneliness, emotional dysregulation, daytime sleepiness) are frequently experienced by both poor sleepers (Griffin et al., 2020; Tucker et al., 2010) and binge‐watchers (Starosta et al., 2021).

We can assume that these factors could cause a pathological chain reaction. In particular, the consequences of sleep disruption in poor sleepers may represent risk factors for the onset of problematic binge‐watching symptoms and motivations, and vice versa. In other words, poor sleepers may be more likely to engage in binge‐watching behaviour due to their greater difficulties in emotional regulation and feelings of loneliness. As a result, binge‐watching may worsen their sleep quality and cause daytime sleepiness, with a negative impact on interpersonal and emotional regulation.

Future studies should conduct targeted investigations to better understand the complex relationship between these factors. In fact, identifying the role of psychological and sleep‐related variables could lead to the development of interventions for the heavy binge‐watcher with low sleep quality. Intervening directly on the factors that play a key role in the onset and maintenance of problematic binge‐watching, such as emotional dysregulation and loneliness, could be a promising therapeutic strategy.

As regards maladaptive motives behind binge‐watching, we also observed a predictive role of male gender and alcohol frequency. In particular, males showed higher scores on the WTSMQ‐coping/escapism dimension. This finding may appear in contrast with past studies that described a correlation between the female gender and binge‐watching frequency and intensity (Pittman & Sheehan, 2015; Spruance et al., 2017). However, the debate on gender prevalence is still open and primarily dependent on the specific type of TV content (Wühr et al., 2017), not explored in the present investigation.

Surprisingly, alcohol frequency during the last year was negatively associated with pathological motives of binge‐watching, and smoking frequency was not significantly associated. Even this result could appear in contrast with previous evidence of a positive association between typical addictive behaviours and binge‐watching aspects (Flayelle et al., 2019a). Nevertheless, we should first consider that our sample was mostly composed of occasional drinkers. Furthermore, this result is in line with a broader perspective on the binge‐watching phenomenon, which is not necessarily related to addictive tendencies.

Despite young people aged 18–39 years account for ~70% of the binge‐watchers in the general population (Starosta & Izydorczyk, 2020), we did not find any association between problematic binge‐watching and age. However, we should bear in mind that our sample is not fully representative of the general population. In fact, it is mainly made up of young adults aged <40 years (90%).

The present study pointed out that the negative aspects of binge‐watching are also associated with different patterns of sleep quality. Furthermore, our findings strengthened the well‐known role of sociodemographic, psychological and sleep‐related characteristics in poor sleepers. Most importantly, our results suggested the existence of a vicious circle that exacerbates the problematic aspects of this practise through the interaction between psychological and sleep‐related factors.

As a whole, we emphasise the importance of not considering binge‐watching as a unitary phenomenon, as it represents a normative way of consuming TV content from one side and a problematic behaviour from another side. The inconsistent evidence in the literature on the association between media bingeing and its consequences on health status could be due to this misleading consideration of binge‐watching as a one‐dimensional phenomenon. Further, we aimed at highlighting the importance of psychological and sleep‐related factors (i.e., loneliness, emotional dysregulation, and daytime sleepiness) behind the problematic dimension of this behaviour.

It is also worth mentioning that our data were acquired during a period (December 2020–January 2021) characterised by a partial regional lockdown imposed by the Italian government (the second contagion wave started in Autumn 2020). Many studies have consistently shown that the restraining measures are associated with increased sleep problems and mental health disturbances amongst the general population (Jahrami et al., 2022; Robinson et al., 2022; Scarpelli et al., 2022). Further, lockdown‐related conditions also affect recreational activities such as binge‐watching behaviour (Boursier et al., 2021; Sigre‐Leirós et al., 2022). Accordingly, our findings may be partly affected by the peculiar condition represented by the lockdown. For example, the high proportion of poor sleepers in our sample (~45%) and the expression of psychological symptoms, such as loneliness and emotional dysregulation, may be driven by these unprecedented circumstances. Likewise, the unexpected results (i.e., predictive role of male gender and alcohol frequency) may be due to the gendered lifestyle changes during the pandemic (Pollard et al., 2020; Rodriguez et al., 2020). Anyway, caution is needed in generalising these findings to the pre‐pandemic period, given the extraordinary context due to the coronavirus disease 2019 (COVID‐19).

From a methodological standpoint, this study has the advantage of using validated and targeted questionnaires to investigate different aspects of the phenomenon. However, some limitations should be mentioned. Firstly, the cross‐sectional design is not informative about any causal relationship between the observed variables. Secondly, the convenience sampling of participants may prevent the generalisation of results to the general population. In addition, although we have adopted some restrictive inclusion criteria (i.e., no assumption of drugs interfering with sleep, no presence of sleep disturbance or mental disease) to exclude possible confounding variables, the survey was self‐administered, and this aspect may introduce a relative bias. Finally, we have no information about the specific time slot of binge‐watching for each participant, which could significantly impact subsequent sleep. Also, the lack of information about the average time spending watching TV series represents a significant limitation and deserves to be considered in future studies to better describe its relationship with different sleep aspects. Longitudinal and follow‐up studies exploring the relationship between sleep‐related factors and different aspects of binge‐watching should be conducted to shed further light on this constantly growing phenomenon.

Especially in light of the steep increase of this compulsive behaviour during the actual COVID‐19 pandemic, an in‐depth understanding of this multifaceted phenomenon becomes even more compelling to develop effective prevention and early intervention where it is necessary. Conversely, an all‐encompassing approach would lead to the risk of overestimating the actual danger of a phenomenon that is so widespread and often harmless nowadays.

## AUTHOR CONTRIBUTIONS

VA, GV, SS, LDG, AM and CF conceivedand designed the experiment; VA, GV, SS and MF collected and pre‐processed thedata; VA, GV, SS and MG analyzed the data; VA, GV, AM, CF and LDG wrote the paper.

## CONFLICT OF INTEREST

None

## Supporting information


**Table S1** Results of the multiple regression model for psychological and sleep‐related variables predicting BWESQ‐binge‐watching scale in poor sleepers (*n* = 200). Asterisks indicate significant predictors
**Table S2** Results of the multiple regression model for psychological and sleep‐related variables predicting WTSMQ‐coping/escapism scale in poor sleepers (*n* = 200). Asterisks indicate significant predictors

## Data Availability

The data that support the findings of this study are available from the corresponding author upon reasonable request.
