# Supplementary material for: ‘This is the last episode’: the association between problematic binge‐watching and loneliness, emotion regulation, and sleep‐related factors in poor sleepers
Source: J Sleep Res. 2022 Oct 17;32(1):e13747. doi: 10.1111/jsr.13747 (PMC10078456; doi:10.1111/jsr.13747)
Supplement: Supplementary file 1 — Table S1 Results of the multiple regression model for psychological and sleep‐related variables predicting BWESQ‐binge‐watching scale in poor sleepers (n = 200). Asterisks indicate significant predictors Table S2 Results of the multiple regression model for psychological and sleep‐related variables predicting WTSMQ‐coping/escapism scale in poor sleepers (n = 200). Asterisks indicate significant predictors [file JSR-32-0-s001.docx]

**Supplementary tables**

**Table S1.** Results of the multiple regression model for psychological and sleep-related variables predicting BWESQ-binge-watching scale in poor sleepers (n=200). Asterisks indicate significant predictors.

| BWESQ-binge-watching scale | β | t  (*p*) | R^2^ | F  (*p*) |
| --- | --- | --- | --- | --- |
|  |  |  | 0.173 | 8.098  (<0.001)** |
| DERS | 0.280 | 3.820  (<0.001) ** |  |  |
| UCLA | 0.152 | 2.157  (0.032) * |  |  |
| ISI | 0.001 | 0.021  (0.983) |  |  |
| MEQr | -0.113 | -1.667  (0.097) |  |  |
| ESS | 0.085 | 1.299  (0.195) |  |  |

Abbreviation: BWESQ, Binge-Watching Engagement and Symptoms Questionnaire; DERS, Difficulties in Emotion Regulation Scale; UCLA, UCLA Loneliness Scale; ISI, Insomnia Severity Index; MEQr, Morningness–Eveningness Questionnaire– reduced version; ESS, Epworth Sleepiness Scale.

**Table S2.** Results of the multiple regression model for psychological and sleep-related variables predicting WTSMQ-coping/escapism scale in poor sleepers (n=200). Asterisks indicate significant predictors.

| WTSMQ-coping/escapism scale | β | t  (*p*) | R^2^ | F  (*p*) |
| --- | --- | --- | --- | --- |
|  |  |  | 0.253 | 13.131  (<0.001) ** |
| DERS | 0.322 | 4.621  (<0.001) ** |  |  |
| UCLA | 0.204 | 3.046  (0.003) ** |  |  |
| ISI | 0.001 | 0.018  (0.986) |  |  |
| MEQr | -0.120 | -1.859  (0.064) |  |  |
| ESS | 0.133 | 2.133  (0.034) * |  |  |

Abbreviation: WTSMQ, Watching TV Series Motives Questionnaire; DERS, Difficulties in Emotion Regulation Scale; UCLA, UCLA Loneliness Scale; ISI, Insomnia Severity Index; MEQr, Morningness–Eveningness Questionnaire– reduced version; ESS, Epworth Sleepiness Scale.
